# Unmasking Idiopathic Brugada ECG Pattern: Inducible Type 1 Brugada Pattern in a Young Patient and Clinical Implications

**DOI:** 10.7759/cureus.40739

**Published:** 2023-06-21

**Authors:** Hajar EL Ouartassi, Badre El Boussaadani, Raid Faraj, Ibtissam Fellat, Mohamed Cherti

**Affiliations:** 1 Cardiology, Ibn Sina University Hospital Center/Mohammed V University Rabat, Rabat, MAR; 2 Cardiology, Mohammed VI University Hospital Center of Tangier/Abdelmalek Essaadi University, Tangier, MAR

**Keywords:** brugada ecg pattern, syncope, ajmaline test, cardiac sudden death, inherited channelopathy, brugada syndrome

## Abstract

Brugada syndrome is a rare inherited channelopathy associated with an increased risk of ventricular tachycardia and ventricular fibrillation, leading to syncope and sudden cardiac death. We present a case report of a young patient with an inducible type 1 Brugada pattern on an electrocardiogram (ECG), accompanied by a comprehensive literature review. The 19-year-old patient presented with dizziness and exhibited a type 2 Brugada pattern on admission ECG, which converted to a type 1 pattern following an Ajmaline test. Based on the absence of symptoms, inducible arrhythmias, or cardiac events in the patient's history, implantable cardioverter-defibrillator insertion was deemed unnecessary. Genetic testing was recommended, and screening ECGs were advised for the patient's first-degree relatives. The discussion explores the different types of Brugada patterns, their diagnostic significance, and the controversies surrounding risk stratification and management strategies. The case underscores the importance of maintaining clinical suspicion for Brugada syndrome in young patients and tailoring treatment approaches based on individual characteristics and risk factors.

## Introduction

Formally known as “Lai Tai” in Thailand, which translates to “death during sleep,” and “Pokkuri” in Japan, indicating “sudden and unexpectedly ceased phenomena” [1], Brugada syndrome is an inherited channelopathy highly prevalent in Southeast Asia. It is associated with an increased risk of ventricular tachycardia (VT) and ventricular fibrillation (VF), leading to syncope and sudden cardiac death (SCD). The syndrome is named after the Brugada brothers, who first described it in 1992 as an ECG abnormality with a high incidence of SCD in patients with structurally normal hearts. Brugada syndrome has an estimated prevalence of 0.1%-0.7% in the adult population and is even rarer in children (0.01%). It predominantly affects males, with a male-to-female ratio of 10:1 [2]. There are two main challenges associated with this electrocardiographic anomaly. Firstly, the electrocardiographic appearance can vary over time within the same patient. Secondly, assessing the risk of SCD is difficult, particularly in asymptomatic patients. In this article, we present a case of a young patient with an inducible type I Brugada ECG pattern, accompanied by a concise literature review.

## Case presentation

**Figure 1:**
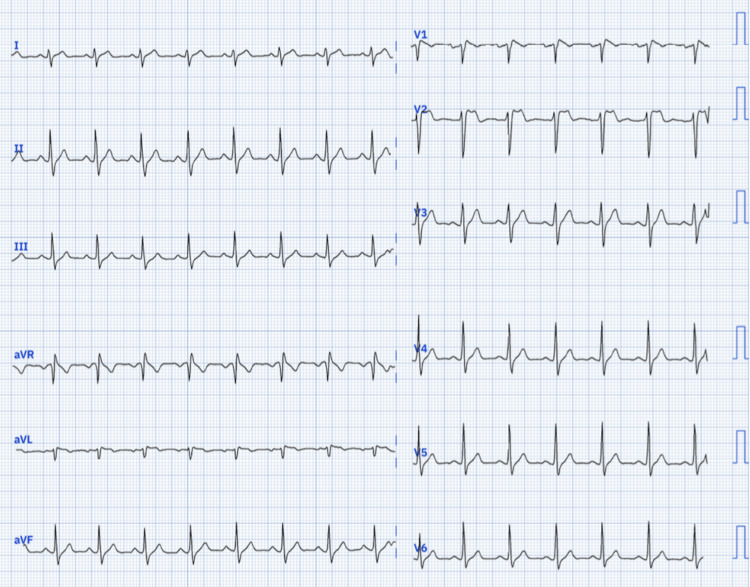
Baseline ECG showing a type 2 Brugada pattern with a saddleback appearance in V2.

A 19-year-old male patient of Indian origin, with no significant personal or family medical history, including syncope or SCD, presented to our department due to an episode of dizziness. Physical examination findings were unremarkable, except for a body temperature of 38.5°C. An ECG performed upon admission revealed a saddleback-shaped ST elevation greater than 2 mm in the right precordial leads, consistent with a type 2 Brugada pattern (Figure 1).

**Figure 2:**
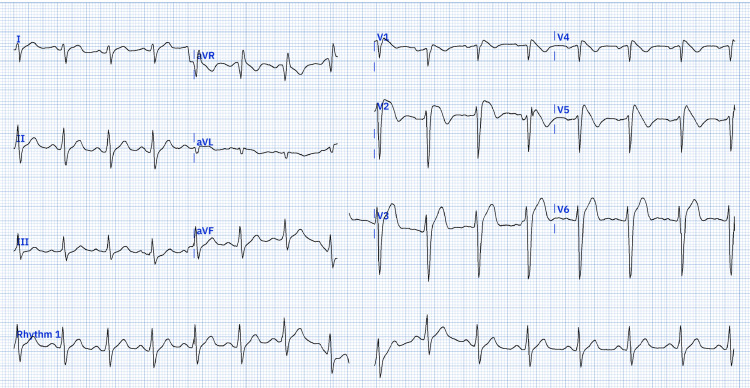
ECG at minute 10 of IV Ajmaline infusion (75 mg) showing a type 1 Brugada pattern with coved ST-segment elevation in right precordial leads.

To further evaluate the patient, an Ajmaline test was conducted, which resulted in the conversion of the type 2 Brugada pattern to a type 1 Brugada pattern (Figure 2).

Subsequently, programmed electrical stimulation (PES) was performed to assess the potential benefits of implanting an implantable cardioverter-defibrillator (ICD), which did not induce any ventricular arrhythmias. The Shanghai scoring system for the diagnosis of Brugada syndrome yielded a score of 2 points, indicating a possibility of Brugada syndrome. Following the evaluation, the patient was discharged with instructions to avoid alcohol and medications with sodium channel-blocking activity. Additionally, the patient was informed about the potential exacerbation of arrhythmias in Brugada syndrome during episodes of fever. Genetic testing was recommended for the patient, and screening electrocardiograms (ECGs) were advised for his first-degree relatives.

## Discussion

Brugada syndrome is categorized into three types based on the ECG repolarization patterns observed in the right precordial leads. Type 1 is characterized by a coved ST segment elevation exceeding 2 mm in leads V1-V3, followed by a negative, symmetric T wave. When specific clinical criteria are present, such as syncope, nocturnal agonal respiration, documented VF, polymorphic VT, inducibility of VT with programmed electrical stimulation (PES), family history of SCD in individuals younger than 45 years, or the presence of similar ECG patterns in family members, this abnormality is considered potentially indicative of Brugada syndrome [3]. Type 2 demonstrates a saddleback-shaped ST elevation greater than 2 mm, with the terminal portion of the ST segment positioned 1 mm above the baseline. Type 3 may exhibit morphology resembling either type 1 or type 2, but the ST segment elevation is less than 2 mm. While types 2 and 3 are not specific enough to be considered diagnostic [4], they can evolve into type 1 spontaneously, in response to fever, or due to certain medications. Diagnostic challenge tests, typically utilizing intravenous Ajmaline, Procainamide, Flecainide, or Pilsicainide, are employed to provoke this transition. 

The role of PES, its necessity, and its impact on the timing of ICD placement in symptomatic patients exhibiting the type 1 ECG pattern, either spontaneously or following sodium channel blockade, remain subjects of debate. The literature reflects varying perspectives on the threshold for conducting PES studies and ICD insertion, with some advocating for a very low threshold, as proposed by Brugada et al., while others support more conservative approaches [5]. In our presented case, an ajmaline challenge resulted in a conversion from a type 2 to a type 1 Brugada pattern. Additionally, no ventricular arrhythmias were induced during programmed electrical stimulation. Considering the absence of a cardiac arrest, tachyarrhythmia, or syncope in the patient's medical history, the insertion of an ICD was deemed unnecessary. Our decision aligns with stratification data suggesting that an inducible type 1 Brugada pattern in asymptomatic patients without prior symptoms or inducible arrhythmias carries a low risk of developing ventricular arrhythmia or SCD (0.3% per year according to the FINGER registry) [6-8]. However, genetic testing was recommended for our patient, and his first-degree relatives were advised to undergo evaluation for the syndrome. Furthermore, the patient was instructed to avoid alcohol and medications that inhibit cardiac sodium channels, such as class I antiarrhythmics, tricyclic antidepressants, and lithium, due to their potential to trigger arrhythmias. Prompt treatment of fevers with antipyretics was emphasized as an important preventive measure for arrhythmias in patients with Brugada syndrome.

## Conclusions

Our case report highlights the significance of maintaining a suitable level of suspicion for Brugada syndrome in young patients. A guideline-based approach helped us to investigate our patients. Although there is much controversy surrounding the risk stratification and therefore the management of certain Brugada patients, physicians should guide their therapeutic strategy according to the symptomatology, history of tachyarrhythmias, and ECG findings before and after a drug challenge.
